# An adolescent presenting with malignant fibrous histiocytoma of the testis: a case report

**DOI:** 10.1186/1752-1947-7-30

**Published:** 2013-01-24

**Authors:** Lian-Li Wang, Hui Xie, Hai-Long Fu, Sen Jiang, Xiu-Fang Wang, Mei-Zai Jia, Ze-Hong Liu, Ya-Ping Zhao

**Affiliations:** 1 Department of Clinical Laboratory, The 82nd Hospital of the People’s Liberation Army, Huaian, 223001, China; 2 Department of Oncology, The 82nd Hospital of the People’s Liberation Army, Huaian, 223001, China

**Keywords:** Testis, Malignant fibrous histiocytoma, Histopathology

## Abstract

**Introduction:**

Malignant fibrous histiocytoma is a very common subtype of soft-tissue sarcoma in middle and late adulthood. However, malignant fibrous histiocytoma of the testis is very rare in adolescents.

**Case presentation:**

We report here the case of a 14-year-old Han Chinese boy, who presented with left scrotal mass lasting for 20 days along with distending pain for 5 days. A physical examination revealed a chicken egg-sized, firm, well-defined mass and unclear epididymis. A B-scan ultrasonography of the left scrotum displayed a 9.0×5.2×4.5cm medium- or low-echoic lobulated mass, which suggested a left testicular neoplasm. A fine needle aspiration cytology examination revealed that the cells obtained from the patient’s testicular neoplasm were composed of myxoid spindle, and ovoid cells with nuclear atypia and mitotic activity, and arranged in a whirlpool or storiform pattern. Under histological examination, the tumor cells were arranged in a storiform pattern, which displayed mucoid matrix degeneration, and grew invasively. Consequently, a histopathological diagnosis suggested myxofibrosarcoma (or myxoid malignant fibrous histiocytoma).

**Conclusions:**

An ultrasonic examination combined with fine needle aspiration cytology should be helpful for the initial differential diagnosis of testicular malignant fibrous histiocytoma. However, the final confirmation relies on histopathological examination. To the best of our knowledge, this is the first reported case of malignant fibrous histiocytoma of the testis in an adolescent.

## Introduction

Malignant fibrous histiocytoma (MFH) is a very common subtype of soft-tissue sarcoma in middle and late adulthood, which usually occurs in the extremities, trunk, and retroperitoneum, and occasionally in the heart, kidney, face, urinary tract, and larynx. It has been reported that MFH is localized to the spermatic cord and epididymis. There are only a few reported cases of MFH in the testicles. Here we present the case of a 14-year-old male adolescent patient with MFH of the testicle discovered by fine needle aspiration (FNA) cytology and confirmed by a histopathological examination.

## Case presentation

**Figure 1:**
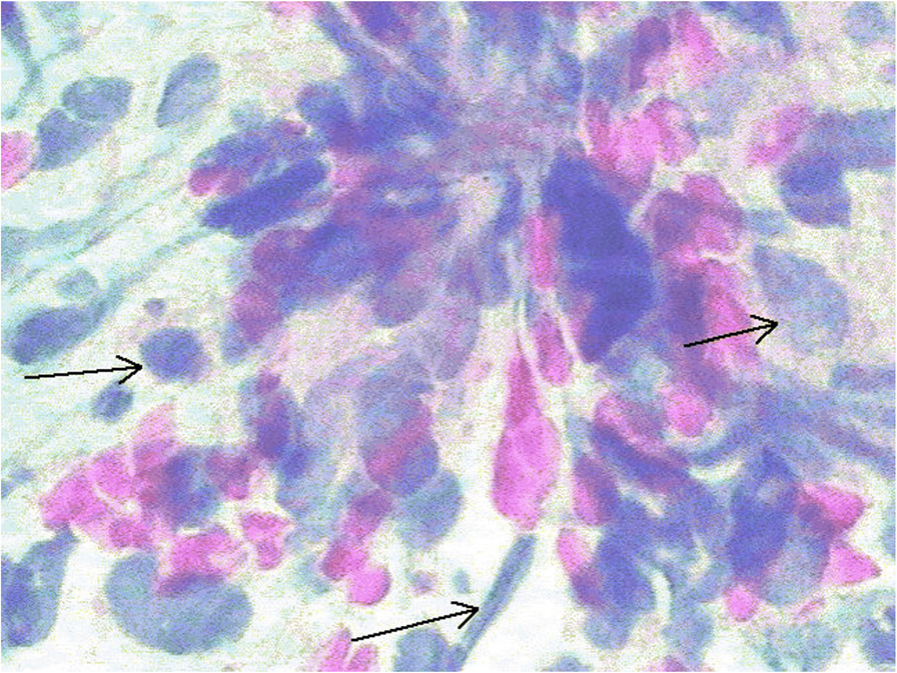
Fine needle aspiration cytology revealed that the cells obtained from testicular neoplasm were myxoid spindle, short spindle or ovoid with nuclear atypia and mitotic activity (arrows) (hematoxylin and eosin, ×400).

**Figure 2:**
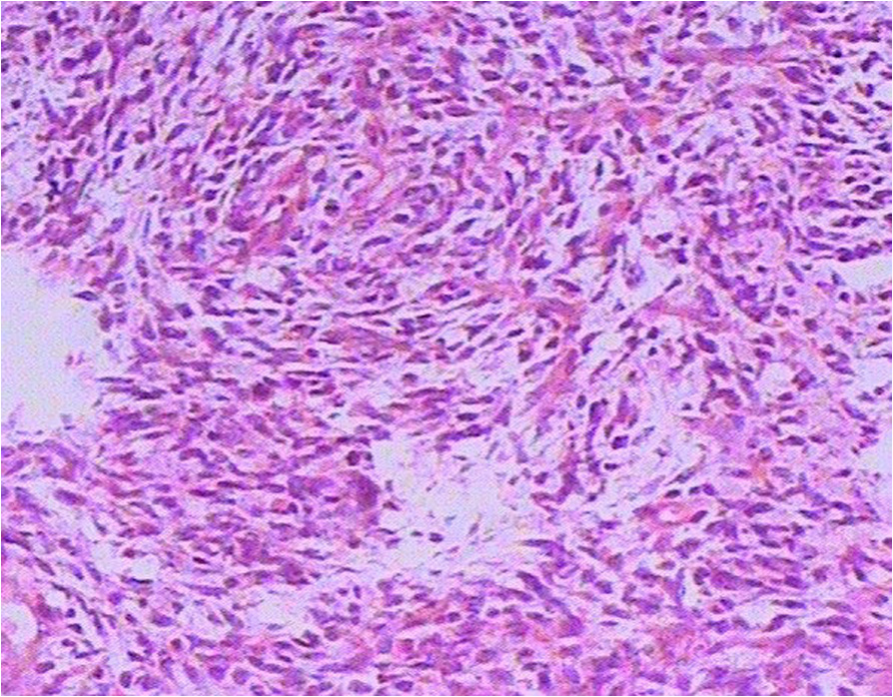
Histopathology of testicular tumor showed spindle, round or ovoid cells arranged in a whirlpool pattern and the scattered multinucleated giant cells in the location of the lesion and the inflammatory cells in the mesenchyma (A) (hematoxylin and eosin, ×100).

**Figure 3:**
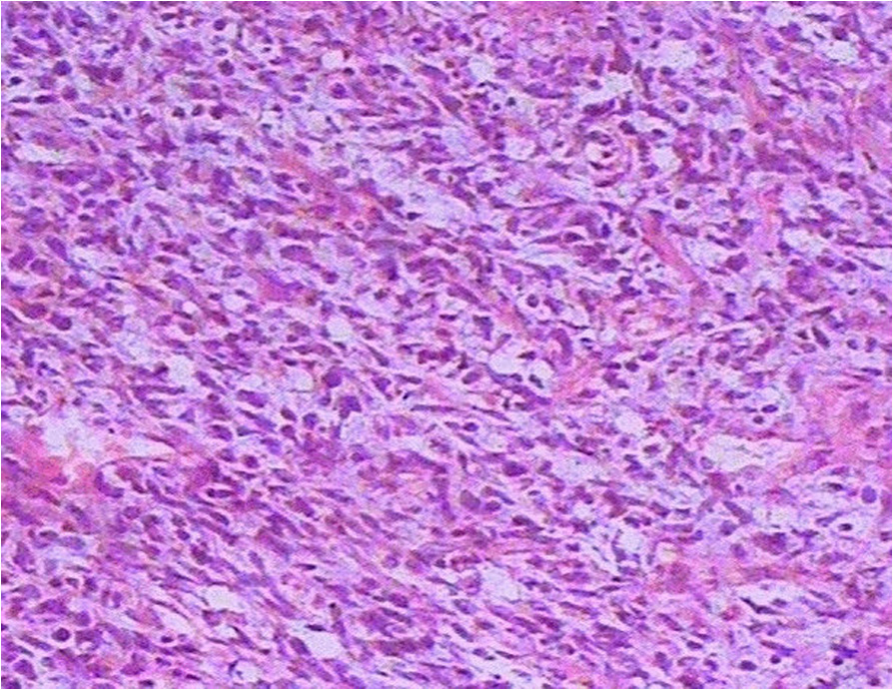
Histopathology of testicular tumor showed spindle, round or ovoid cells arranged in storiform pattern. and the scattered multinucleated giant cells in the location of the lesion and the inflammatory cells in the mesenchyma (B) (hematoxylin and eosin, ×100).

A 14-year-old Han Chinese boy was admitted to our hospital because of left scrotal mass lasting for 20 days along with distending pain in scrotum for 5 days. A physical examination revealed a chicken egg-sized, firm, well-defined tender mass and unclear epididymis. The spermatic cord was felt to be thicker with tenderness. No lymph nodes were detected in bilateral inguen. A B-scan ultrasonography of the left scrotum displayed a 9.0×5.2×4.5cm medium- or low-echoic lobulated mass, which suggested a left testicular neoplasm. A FNA cytology examination revealed that the cells obtained from the patient’s testicular neoplasm were composed of myxoid spindle, and ovoid cells with nuclear atypia and mitotic activity, arranged in a whirlpool or storiform pattern (Figure 1), which suggested a malignant tumor. The patient underwent a left radical orchiectomy. At surgery, a 10.5×5.0×4.5cm, well-encapsulated mass was found in the patient’s left testicle and the mass was dissected and removed. By macroscopic examination of the resected specimen we observed many blood vessels distributed across the surface of the mass, and the section of the mass was solid, lobulated, grayish-yellow and tender. A histopathological examination revealed that the tumor cells displayed spindle, round or ovoid shapes with mitotic activity and mucoid matrix degeneration, and they were arranged in a whirlpool or storiform pattern and grew invasively. Multinucleated giant cells were scattered in the location of the lesion. Infiltration of the inflammatory cells was seen in the mesenchyma (Figures 2 and 3). All these histopathological features are consistent with myxoid MFH.

## Discussion

Testicular tumors, which account for 1% to 2% of male tumors, constitute a small proportion of malignancies. Testicular MFH is a rare disease, especially in adolescents. To the best of our knowledge, of the three cases of testicular MFH that were reported in the literature, one case was in English and the other two were in Chinese, and the patients’ ages ranged from 56 to 78 years old [1-3]. According to a review of the literature, ours is the first reported case of testicular MFH in an adolescent.

At the initial stage of the testicular MFH, the patient had no obvious symptoms or signs and exhibited a gradually enlarging mass in his scrotum. Owing to the growing tumor, the patient felt severe distending pain in his scrotum in the advanced stage. MFH is an aggressive tumor with poor prognosis. Surgical resection is currently the first therapeutic approach of testicular MFH. After surgery, the patient was treated with adjuvant radiotherapy and chemotherapy. However, according to the literature, postoperative adjuvant radiotherapy and chemotherapy have an unclear effect on the reduction of local recurrence and distant metastasis [4]. Our adolescent patient died from lung metastasis 6 months after the operation.

MFH is composed of histiocyte-like and fibroblast-like cells arranged in a storiform pattern and accompanied by pleomorphic cells, and multinucleated giant cells [5]. MFH has been divided into five subtypes: myxoid, angiomatoid, giant cell, inflammatory, and storiform-pleomorphic [6]. Based on the new World Health Organization classification of soft tissue tumors introduced in late 2002, many of the above-mentioned MFH subtypes have been changed. MFH is now divided into five subtypes: myxofibrosarcoma, angiomatoid fibrous histiocytoma, undifferentiated pleomorphic sarcoma with giant cells, undifferentiated pleomorphic sarcoma with prominent inflammation, and undifferentiated high-grade pleomorphic sarcoma. In this case, the testicular MFH belongs to the myxofibrosarcoma subtype.

It is difficult to diagnose MFH only by imaging methods, such as ultrasound, computed tomography and magnetic resonance imaging (MRI). The definitive diagnosis is often a diagnosis of exclusion confirmed by histological analysis [7,8]. In this case, an ultrasound examination exhibited a solid mass in the patient’s testicles. Subsequently, FNA cytology showed a malignant tumor. Finally, a histological examination revealed classification of the testicular MFH as a myxofibrosarcoma subtype, which was in accord with the FNA cytology. We consider that the FNA cytology played a key role in the early diagnosis of the malignant tumor.

## Conclusions

In conclusion, our case demonstrates that testicular MFH may occur in an adolescent, and it seems to be more malignant because our patient survived only 6 months. Ultrasonic examination combined with FNA cytology should be helpful for the initial differential diagnosis of testicular MFH. However, the final confirmation relies on histopathological examination. The molecular methodology (for example protein expression and/or microribonucleic acid (microRNA) and/or epigenetic modification) might provide new insight into MFH.

## Consent

Written informed consent was obtained from the patient’s legal guardian, his grandfather, for publication of this case report and accompanying images. A copy of the written consent is available for review by the Editor-in-Chief of this journal.

## Competing interests

The authors declare that they do not have any competing interests.

## Authors’ contributions

LLW and HX were major contributors in writing the manuscript; YPZ and HX analyzed and interpreted the patient’s data regarding the MFH disease. LLW, HLF, SJ, XFW, MZJ and ZHL performed the FNA cytology and histological examination of the testis. All authors read and approved the final manuscript.
